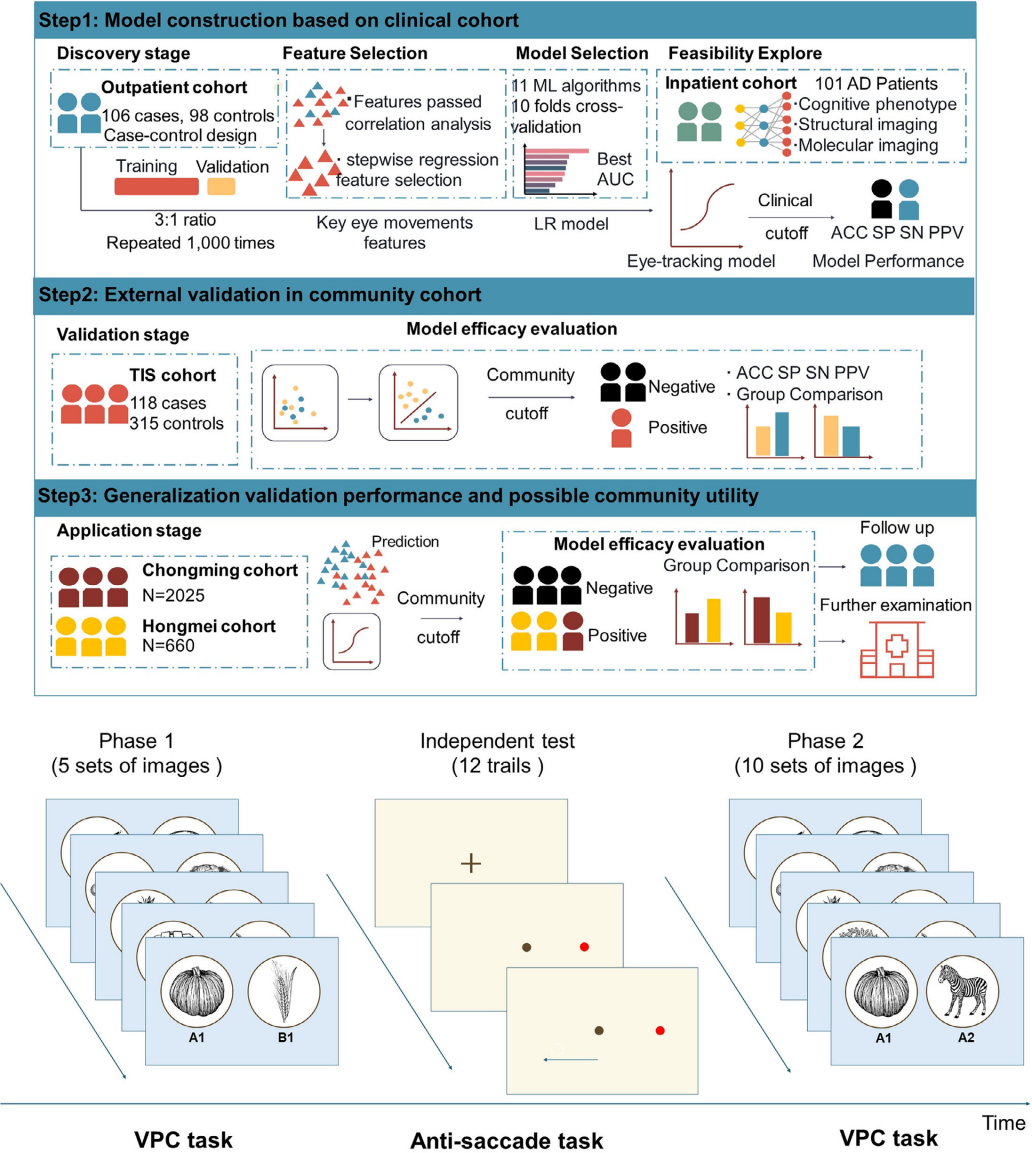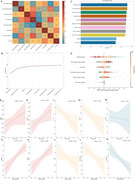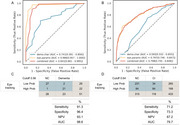# Artificial intelligence‐based eye‐tracking measurement for early detecting cognitive impairment

**DOI:** 10.1002/alz70856_101608

**Published:** 2025-12-24

**Authors:** Mingxia Wei, Jincheng Li, Nathan Zhao, Yingzhe Wang, Qiang Dong, Yanfeng Jiang, Mei Cui

**Affiliations:** ^1^ Huashan Hospital, Fudan University, Shanghai, Shanghai, China; ^2^ Fudan University Taizhou Institute of Health Sciences, Taizhou, China; ^3^ Human Phenome Institute, Fudan University, Shanghai, Shanghai, China; ^4^ Human Phenome Institute, Fudan University, Shanghai, China; ^5^ State Key Laboratory of Genetic Engineering, Human Phenome Institute, Zhangjiang Fudan International Innovation Center, Fudan University, Shanghai, Shanghai, China; ^6^ Fudan University Taizhou Institute of Health Sciences, Taizhou, Jiangsu, China; ^7^ MOE Frontiers Center for Brain Science, Fudan University, Shanghai, China

## Abstract

**Background:**

With the aging global population, timely detection of cognitive impairment (CI) is key to reduce the burden of dementia. However, early diagnosis of CI in China is challenging due to time‐consuming, subjective neuropsychological assessments, especially in low‐resource areas. Digital biomarkers, such as eye movement features, offer promising potential due to their accessibility. To address this, we developed an AI‐based eye‐tracking system using a portable tablet, offering an efficient, objective, and scalable solution for CI detection, with the goal of establishing a feasible CI management framework as a government priority.

**Method:**

Data were analyzed from the outpatient individuals (*N* = 204) and inpatient individuals (*N* = 101) and three community cohorts, including the Taizhou Imaging Study (TIS) (*N* = 443), Hongmei (*N* = 660) and Chongming cohort (*N* = 2025). Firstly, we selected eye movement features and optimal machine learning algorithms and then conducted the eye‐tracking model in outpatient individuals. Associations were analyzed between eye movement features, cognitive phenotype, structural imaging, and molecular imaging in inpatient participants. Additionally, the model was evaluated in TIS and further evaluated for potential community utilities in Chongming and Hongmei cohort.

**Result:**

For detecting CI, the eye‐tracking model, which incorporates six eye movement features based on logistic regression, achieved an area under the receiver operating curve (AUC) of 0.986, SN of 91.3%, SP of 96.4%, NPV of 93.1% in internal validation in outpatient individuals. The selected features were strongly associated with neuropsychological scores and brain atrophy scores in inpatient participants. Notably, the central(mean) features showed a significant negative association with tau deposition in the temporal lobe (*p* = 0.05). For discriminate CI in community settings, the eye‐tracking model achieved an AUC of 0.797, SN of 71.2% and SP of 73.3%, NPV of 87.2% in the external validation in TIS cohort. To evaluate the risk generalization ability of the eye‐tracking model, individuals classified as high probability (with prediction probabilities exceeding the cutoff) showed significantly higher AD8 scores (*p* = 0.002 for Hongmei, *p* <0.001 for Chongming).

**Conclusion:**

The AI‐based eye‐tracking system can serve as a potential method for preliminarily screening for CI to improve early diagnosis, prompt treatment and rational allocation of medical resources.